# Lists with and without Syntax: A New Approach to Measuring the Neural Processing of Syntax

**DOI:** 10.1523/JNEUROSCI.1179-20.2021

**Published:** 2021-03-10

**Authors:** Ryan Law, Liina Pylkkänen

**Affiliations:** ^1^NYUAD Institute, New York University Abu Dhabi, Abu Dhabi, United Arab Emirates; ^2^Department of Psychology, New York University, New York, New York 10003; ^3^Department of Linguistics, New York University, New York, New York 10003

**Keywords:** magnetoencephalography, semantics, syntax, word lists

## Abstract

In the neurobiology of syntax, a methodological challenge is to vary syntax while holding semantics constant. Changes in syntactic structure usually correlate with changes in meaning. We approached this challenge from a new angle. We deployed word lists—typically, the unstructured control in studies of syntax—as both test and control stimuli. Three-noun lists (“lamps, dolls, guitars”) were embedded in sentences (“The eccentric man hoarded lamps, dolls, guitars…”) and in longer lists (“forks, pen, toilet, rodeo, lamps, dolls, guitars…”). This allowed us to minimize contributions from lexical semantics and local phrasal combinatorics: the same words occurred in both conditions, and in neither case did the list items locally compose into phrases (e.g., “lamps” and “dolls” do not form a phrase). Crucially, the list partakes in a syntactic tree in one case but not the other. Lists-in-sentences increased source-localized MEG activity at ∼250–300 ms from each of the list item onsets in the left inferior frontal cortex, at ∼300–350 ms in the left anterior temporal lobe and, most reliably, at ∼330–400 ms in left posterior temporal cortex. In contrast, the main effects of semantic association strength, which we also varied, localized in the left temporoparietal cortex, with high associations increasing activity at ∼400 ms. This dissociation offers a novel characterization of the structure versus word meaning contrast in the brain: the frontotemporal network that is familiar from studies of sentence processing can be driven by the sheer presence of global sentence structure, while associative semantics has a more posterior neural signature.

**SIGNIFICANCE STATEMENT** Human languages all have a syntax, which both enables the infinitude of linguistic creativity and determines what is grammatical in a language. The neurobiology of syntactic processing has, however, been challenging to characterize despite decades of study. One reason is pure manipulations of syntax are difficult to design. The approach here offers a novel control of two variables that are notoriously hard to keep constant when syntax is manipulated: word meaning and phrasal combinatorics. The same noun lists occurred inside longer lists and sentences, while semantic associations also varied. Our MEG results show that classic frontotemporal language regions can be driven by sentence structure even when local semantic contributions are absent. In contrast, the left temporoparietal junction tracks associative relationships.

## Introduction

Syntax is a combinatorial system that relates linguistic elements during complex meaning construction. Its neurobiology has been studied extensively for decades, yet a lack of consensus persists (for review, see Kaan and Swaab, 2002; Hagoort, 2014; Friederici, 2017; Pylkkänen, 2019; Matchin and Hickok, 2020). One likely reason is a principled methodological challenge: it is very difficult to vary the syntactic structure of an expression without also altering its compositional semantics. Consequently, the nature and even existence of purely structural processing in the brain remains elusive. Here, we introduce a new experimental manipulation that succeeds in controlling certain semantic variables: specifically, word meaning and local semantic composition. With these robust modulators of neural activity controlled, will correlates of purely structural processing emerge?

**Figure 1.**
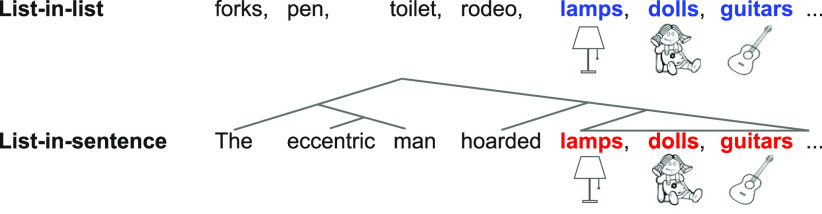
A schematic depiction of our structure contrast. The same noun list is embedded in a longer list (list-in-list) and in a sentence (list-in-sentence).

Our study exploited the fact that word lists—typically used as unstructured control stimuli in studies of syntax—can also naturally occur inside a sentence, participating in the syntax of the sentence. For example, identical noun sequences can occur in longer lists and in sentences (Fig. 1). In this contrast, the embedded three-noun lists across the pair of list-in-list and list-in-sentence are matched in at least the following aspects: (1) lexical characteristics (e.g., word form, concreteness, frequency, morphemic structure); and (2) local combinatorics: in neither case do these words semantically or syntactically compose with one another (e.g., “lamps” and “dolls” do not form a phrase). A schematic depiction of this contrast is shown in Figure 1. The “syntactic engine” operates through the lists in sentences but not in longer lists. This is our core contrast.

The literature deploying the sentence versus list paradigm—albeit not in the controlled fashion that we do here—forms a particularly relevant background for the current study. Emerging from this literature is a left lateral “combinatory network” (for review, see Pylkkänen and Brennan, 2019; but also see Ferstl et al., 2008; Fedorenko et al., 2012; Friederici and Gierhan, 2013) that subserves the composition of word meanings into larger syntactic and semantic structures: anterior temporal lobe (ATL), posterior temporal lobe (PTL), inferior frontal cortex (IFC), temporoparietal junction (TPJ), and orbitofrontal cortex (ORB). Summarized in Table 1, the left ATL is the most consistent correlate for sentence structure despite the variability in imaging techniques, stimulus presentation modalities, and types of unstructured controls. Within the context of linguistic meaning composition, targeted research on the ATL using magnetoencephalography (MEG) has, however, shown that the ATL tracks aspects of conceptual combinatorics rather than syntactic structure building (for review, see Pylkkänen, 2019; see also Baron and Osherson, 2011 and Coutanche et al., 2019).

**Table 1.** A summary table showing a number of studies contrasting sentences to word lists using different imaging modalities, stimulus modalities, and control types

Studies	Imaging modality	Stimulus modality	Word list type	Language	ATL	PTL	IFC	TPJ	ORB
Mazoyer et al., 1993	PET	Auditory	Content and function	French	×				
Stowe et al., 1998	PET	Visual	Content and function	Dutch	×				
Vandenberghe et al., 2002	PET	Visual	Sentence scrambled	English	×	×			
Humphries et al., 2005	fMRI	Auditory	Content only	English	×	×			
Humphries et al., 2006	fMRI	Auditory	Sentence scrambled	English	×			×	
Snijders et al., 2009	fMRI	Visual	Content and function	German	×	×	×		
Pallier et al., 2011	fMRI	Visual	Content and function	French	×	×	×	×	
Brennan and Pylkkänen, 2012	MEG	Visual	Content and function	English	×	×	×		×
Fedorenko et al., 2012	fMRI	Visual	Content and function	English	×	×	×	×	
Matchin et al., 2017	fMRI	Visual	Content and function	English	×	×	×		
Zaccarella et al., 2017	fMRI	Visual	Content only	German		×	×		

The current study also used MEG, allowing us to measure reflections of structure position by position within our lists to better understand the temporal nature of the effect. We also included a manipulation along a semantic dimension: association strength between the list members. One possibility is that, regardless of the linguistic context surrounding the noun lists, the brain might (1) compose meaning when co-occurrence statistics between items are sufficiently high (Mollica et al., 2020) and/or (2) more generally “chunk” together nouns into some abstract representations (Christiansen and Chater, 2016; e.g., creating a coherent “grocery bag” scene from “juice,” “tomatoes,” “pasta”). Thus, by varying association strength, we aimed to distinguish potential effects of structure from effects of associative semantics. To reiterate, members of noun lists do not compose with one another, either syntactically or semantically. In this sense, our manipulation of association is different from that of semantic composition.

Neural responses were recorded as participants read word-by-word the same noun lists embedded in longer lists (unstructured controls) and in sentences (structured stimuli) then responded to a memory probe. Behaviorally, we would expect that the presence of structure facilitates recall (Potter et al., 1980, 2008). Neurally, we would expect the presence of structure to elevate cortical activation independent of word meaning if syntactic structure and lexical meaning can be dissociated.

## Materials and Methods

### 

#### Stimuli and design

We selected concrete English nouns based on the concreteness rating corpus by Brysbaert et al. (2014). From this pool, we then selected nouns that are matched in their log frequency from the SUBTLEX-US corpus (Brysbaert and New, 2009). The critical list nouns were changed from their singular form to plural to block potential noun-noun compounding (e.g., “lamp doll” could form a phrase, but “lamps dolls” could not). These plural nouns were then used to construct our critical three-noun lists such as lamps, dolls, guitars. The lexical characteristics are summarized in Table 2.

**Table 2.** Lexical characteristics of the critical items

Condition	Log frequency	Concreteness	Word length	Sentence plausibility	Association strength
List-in-list, low association	2.45 (0.65)	4.87 (0.13)	6.02 (1.68)		0.44 (0.09)
List-in-sent, low association	5.95 (1.40)	0.45 (0.09)			
List-in-list, high association	2.59 (0.63)	4.84 (0.18)	5.56 (1.49)		0.59 (0.10)
List-in-sent, high association	6.18 (1.30)	0.62 (0.09)			

Mean values for each measure are reported, with SD in parentheses.

For lists-in-lists, the nouns surrounding the critical lists were assigned at random. We prepended four and appended three nouns to the critical lists, resulting in 10-word sequences. The number of plural, singular mass, and singular count nouns was balanced. Items from the critical lists were included in other items as noncritical nouns (i.e., filler nouns surrounding critical lists) to balance out the co-occurrence statistics of the critical items within our stimulus set. A reviewer noted that this might result in differences in word familiarity and thus introduce a confound. Repeating the critical nouns may indeed increase word familiarity by increasing their relative frequency in the stimuli. However, word familiarity would increase across all levels of our manipulation. Therefore, we do not think that this is a confound or that it affects our interpretation of the results, even if it were true. As for lists-in-sentences, the same critical lists were given a sentence frame. We prepended a subject and a verb, as well as appended two additional nouns connected with the conjunction and to the end of critical lists, resulting in 10-word sentences. The presence of a determiner preceding the critical lists was balanced across conditions.

For the association strength manipulation, we calculated word co-occurrence statistics by first extracting vectors of the stimulus content words from a pretrained Global Vectors model (Pennington et al., 2014). Then, we calculated the cosine similarity of content words across words 1–7 and made sure that the distribution of association strength was bimodal, with high and low association cases reflecting each of the local maxima. The sentences were then submitted to a norming survey for plausibility on Amazon Mechanical Turk. A stimulus set thus consisted of four 10-word sequences: two lists-in-lists and two lists-in-sentences. Set-hood was defined here by a common word 7 (e.g., “guitars”) instead of words 5 and 6 so as to allow us to vary those words and subsequently varying association strength. Since word interpretations are shifted by context (Nieuwland and Van Berkum, 2006; Kutas and Federmeier, 2011), varying semantic association by calculating the cosine similarity between the context and the target word vectors also allowed us to quantitatively approximate the effects of the sentence-level semantics. An example stimuli set is shown in Table 3. In total, the experiment was composed of 168 trials.

**Table 3.** One complete set of stimuli showing the full 2 × 2 design crossing structure and association

Structure	Association	Words 1–4	Words 5–7	Words 8–10
List-in-list	Low	Forks pen toilet rodeo	Lamps dolls guitars	Wood symbols straps
List-in-sent	Low	The eccentric man hoarded	Lamps dolls guitars	Watches and shoes.
List-in-list	High	Theatre graves drums mulch	Pianos violins guitars	Crates knuckle cocoa
List-in-sent	High	The music store sells	Pianos violins guitars	Drums and clarinets.

#### Experimental procedures

**Figure 2.**
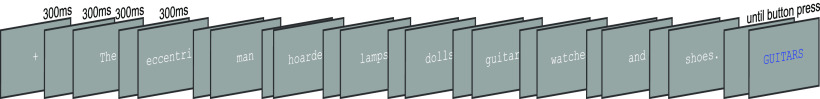
Trial structure.

Stimuli were delivered using rapid serial visual presentation of white text on gray background backprojected onto a monitor ∼80 cm away from participants' heads. Participants initiated each trial via a button press. Each trial began with a fixation cross on screen for 300 ms, followed by an interstimulus interval (ISI) of 300 ms. Stimulus words were also presented on screen for 300 ms then an ISI for 300 ms. At the end of each trial, a memory probe appeared on screen, consisting of a word in blue. Participants responded to the task via button press: they pressed the left button if that word was drawn from that trial, and the right button if not (Fig. 2). Behavioral reaction times and accuracy scores were measured from the presentation of the memory probe task. Items were fully randomized across the experiment.

The memory probe task was selected for both lists-in-lists and lists-in-sentences to monitor participants' attention. A random word (either content or function) was drawn pseudorandomly from each trial. Although the task was likely less demanding for lists-in-sentences, as sentences might be privileged in working memory (Baddeley et al., 2009) and therefore were expected to be easier than lists-in-lists (Potter et al., 1980, 2008), adopting a parallel task across conditions was deemed more important. In fact, under the assumption that harder processing engages the brain to a greater extent, having a word recall task might increase the responses of the brain to the unstructured list-in-list conditions. Furthermore, as previously mentioned, a possibility remains that participants work to create phrases out of word lists or use chunking as a strategy when faced with a difficult task. Together, the experimental conditions might reduce the difference in cognitive operations engaged by the two conditions and thus biasing our study against finding an activity increase for lists-in-sentences over lists-in-lists, which was the effect of interest. No part of the study procedures was preregistered.

#### Participants

Twenty-two native English speakers participated in the experiment. Two were excluded because of technical issues during data acquisition; four were excluded because of excessive sensor noise. Thus, a total of 16 participants were included in our analyses (9 women; mean age = 24.8 years; SD = 7.4 years). All participants are right handed and reported no history of neurologic disorder. The precise sample size was not determined in advance, though our recruitment goal was to achieve a similar sample as in our prior studies on syntactic and semantic processing, ∼20–25 participants (Bemis and Pylkkänen, 2011; Westerlund and Pylkkänen, 2014).

#### Data acquisition and preprocessing

Before recording, each participant's head shape was digitized using a Polhemus FastSCAN system (Polhemus). Digital fiducial points were recorded individually, including three anatomic landmarks (the nasion and the left and right tragi) and five marker coil positions (three points on the forehead and one point each at 1 cm anterior to the left and right tragi). Marker coils were placed at the same five positions to localize the participant's head relative to the MEG sensors. The measurements of head position using marker coils were recorded right before and after experiment to correct for movement during recording. MEG recordings were collected in the MEG Laboratory at New York University Abu Dhabi using a whole-head 208-channel axial gradiometer system (Kanazawa Institute of Technology, Kanazawa, Japan) as participants lay supine in a dimly lit, magnetically shielded room. A practice session first took place outside the magnetically shielded room.

MEG recordings were sampled at 1000 Hz with an online bandpass filter between 0.1 and 200 Hz and noise was reduced using eight reference channels via the Continuously Adjusted Least-Squares Method (Adachi et al., 2001) using the MEG160 Laboratory software (Yokogawa Electric Corporation and Eagle Technology Corporation). The noise-reduced MEG recording, the digitized head shape, and the head position measurements were then imported into MNE-Python (Gramfort et al., 2014). Data were submitted to an offline low-pass filter of 40 Hz with a finite impulse response filter design using a Hamming window method. Flat or excessively noisy channels were interpolated using the spherical spline method (Perrin et al., 1989). The data were then submitted to an independent-component analysis for detection and removal of well characterized artifacts (eye blinks and heart beats) and noise components characteristic of the MEG system. Finally, data were segmented into epochs spanning the whole 10-word sequences, each baselined using the 200 ms period before trial onset. Epochs were automatically rejected if any sensor value after noise reduction exceeded 2.5 pT/cm at any time. Then, epochs were trimmed to contain only the critical list items.

We estimated cortical activity by creating dynamic statistical parameter maps (Dale et al., 2000). First, MEG data were coregistered with either the participant's anatomic MRI when available or the FreeSurfer average brain when not (CorTechs Labs Inc.; and Massachusetts General Hospital/Harvard Medical School/MIT Athinoula A. Martinos Center for Biomedical Imaging, Cambridge, MA). The FreeSurfer average brain was scaled to match the participant's head shape while aligning the fiducial points. Minute manual adjustments were conducted to minimize the difference between the head shape and the average brain. Next, a source space was set up, with each hemisphere containing 2562 potential electrical sources. A forward solution was then computed using the boundary element model. Channel noise covariance matrices were estimated using the baseline period (200 ms before trial started) and regularized using the automated method (Engemann and Gramfort, 2015). Combining the forward solution and noise covariance matrices, an inverse solution was computed and applied to participant-evoked responses assuming a free orientation of the current dipole to yield cortical source activity estimates.

**Figure 3.**
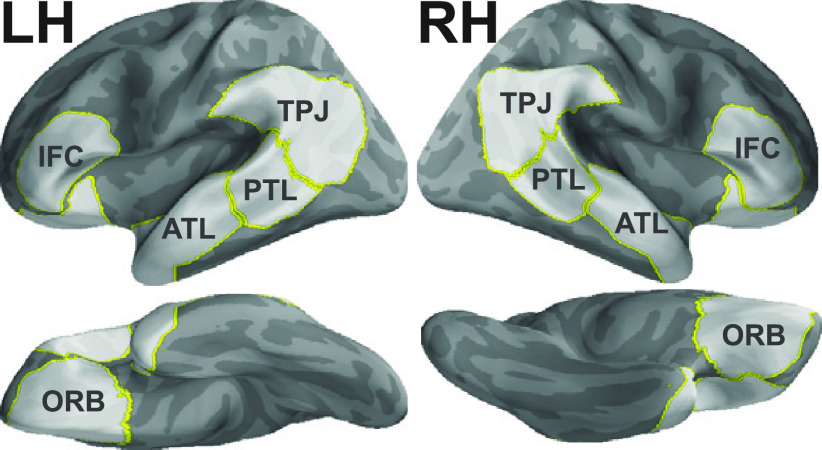
Regions of interest.

Our primary analyses were performed on source activity localized to the five regions of interest (ROIs) from each hemisphere (Fig. 3). The inclusion of right hemisphere homologs was motivated by findings showing right hemisphere involvement during combinatory language understanding (Mazoyer et al., 1993; Stowe et al., 1998; Humphries et al., 2006; Rogalsky and Hickok, 2009). The ROI labels are defined and generated as follows. For the left IFC, ATL, and PTL, 30 mm spheres were created around coordinates for the left inferior frontal cortex, temporal pole, anterior superior temporal sulcus, and posterior superior temporal sulcus in Montreal Neurologic Institute (MNI) space reported by Pallier et al. (2011). We used functional activation peaks from this study because (1) their paradigm contains our main structure contrast, (2) their stimulus presentation resembled ours (word-by-word visual presentation at 300 ms/word), (3) their stimuli contained a similar number of words compared with ours, and (4) the peaks identified are compatible with a large body of literature (for review, see Friederici, 2011, 2017; Hagoort, 2014; Matchin and Hickok, 2020). Spheres for the left temporal pole and anterior superior temporal sulcus were combined, because studies often find these regions coactivating, and because MEG is less spatially resolved compared with fMRI. To generate the right hemisphere homologs of these regions, the polarity of the *x*-axis values in the MNI coordinates were flipped before 30 mm spheres were created around them. Both left and right TPJs were generated by combining the angular gyrus (Brodmann area 39) and adjacent supramarginal gyrus (Brodmann area 40) labels from the “PALS-B12-Brodmann” atlas (Van Essen, 2005), while left and right ORBs were generated by combining lateral and medial orbitofrontal labels from the “aparc” atlas (Desikan et al., 2006).

#### Statistical analysis

##### Behavioral analyses

For each participant, we removed reaction time measures that either corresponded to incorrect responses or were 2 SDs from the participant's own mean. To analyze reaction times, we fitted a linear mixed-effects regression model to log-transformed reaction time data using the lme4 package in R (Bates et al., 2015). The model included fixed effects for structure (list-in-list, list-in-sentence), association (continuous association measures), and an interaction term (structure by association). For random effects, we iteratively reduced model complexity from a maximal model, following Bates et al. (2018). This led to a more parsimonious mixed model with the same fixed effects but only included by-participant varying intercepts, varying structure slopes, with slope/intercept correlations, as well as by-item varying intercepts. Both the maximal and more parsimonious models arrived at the same statistical inference about the fixed effects. Here, we report statistics from the latter. As for accuracy data, we fitted a generalized mixed-effects logistic regression to the accuracy data (incorrect responses included). Also, through model comparison, this model included as random effects a full random-effects structure for all fixed effects, together with by-item varying intercepts.

##### ROI and whole-brain analyses

We performed temporal and spatiotemporal nonparametric cluster-based permutation tests (Maris and Oostenveld, 2007) using the Python package Eelbrain (version 0.30.11; https://zenodo.org/record/2653785) in each ROI and across the whole brain, respectively (Bemis and Pylkkänen, 2011, similar application). For temporal tests, source-localized MEG estimates were first averaged across sources within each ROI. We included word position (words 5, 6, and 7) as a factor. This allowed us to examine potential structure effects time locked to word presentation. Then, a 2 × 2 × 3 repeated-measures ANOVA was fitted at each time sample separately across the whole epoch (600 ms) separately in each ROI. Factors included structure (list-in-list, list-in-sentence), association (high, low), as well as position (word 5, word 6, word 7). Temporal analyses adopted a cluster-forming threshold of *p* < 0.05 with a minimum of 20 contiguous time samples. For the spatiotemporal test, we followed the same procedure, but instead performing a spatiotemporal search across the whole brain (i.e., without averaging activity across source space). In this analysis, an additional criterion of a cluster-forming threshold with at least 20 contiguous spatial samples was adopted. Cluster-level *p* values were first estimated via Monte Carlo simulations, repeated 10,000 times. These *p* values were then corrected for multiple comparisons across all ROIs by controlling the false discovery rate (FDR) at the critical value of 0.05 (Benjamini and Hochberg, 1995). For all permutation tests, we also adopted the threshold-free cluster enhancement procedure (Smith and Nichols, 2009) to obviate the need for a hard-coded cluster-forming threshold and arrived at the same statistical inference about the effects. All cluster tests were not performed separately for each effect.

## Results

### Behavioral results

We found a main effect of structure in both reaction times (χ^2^ = 47.62, *p* < 0.001) and accuracy data (χ^2^ = 20.24, *p* < 0.001). Compared with the corresponding list-in-list conditions, participants were markedly faster and more accurate for lists-in-sentences (mean ± SD: 965 ± 294 ms; 96 ± 18%) relative to lists-in-lists (828 ± 249 ms; 88 ± 31%). Association strength between list items and contexts did not modulate reaction times or accuracy (both *p* > 0.425). There were no interactions between structure and association across both analyses (both χ^2^ < 0.04, *p* > 0.84).

As expected, we found that structure facilitated recall. Here, we explore two possible explanations. First, sentences are much more engaging as stimuli than long lists of nouns in general. Thus, participants might have paid more attention to sentence stimuli, leading to better performance as a result. Second, although both structure types contain the same number of words, the likelihood of drawing a noun is higher in lists-in-lists (which consisted only of nouns) compared with lists-in-sentences. As such, there were more noun competitors for lists-in-lists in the word recall task, which may subsequently reduce response accuracy and increase reaction times. Importantly though, the present behavioral results demonstrate that the word recall task was less demanding for conditions with sentence structure. Under the assumption that an easier task engages the brain to a lesser extent, any increase in our structured stimuli could not be attributed to general increased task effort.

### Neural effects of structure

**Figure 4.**
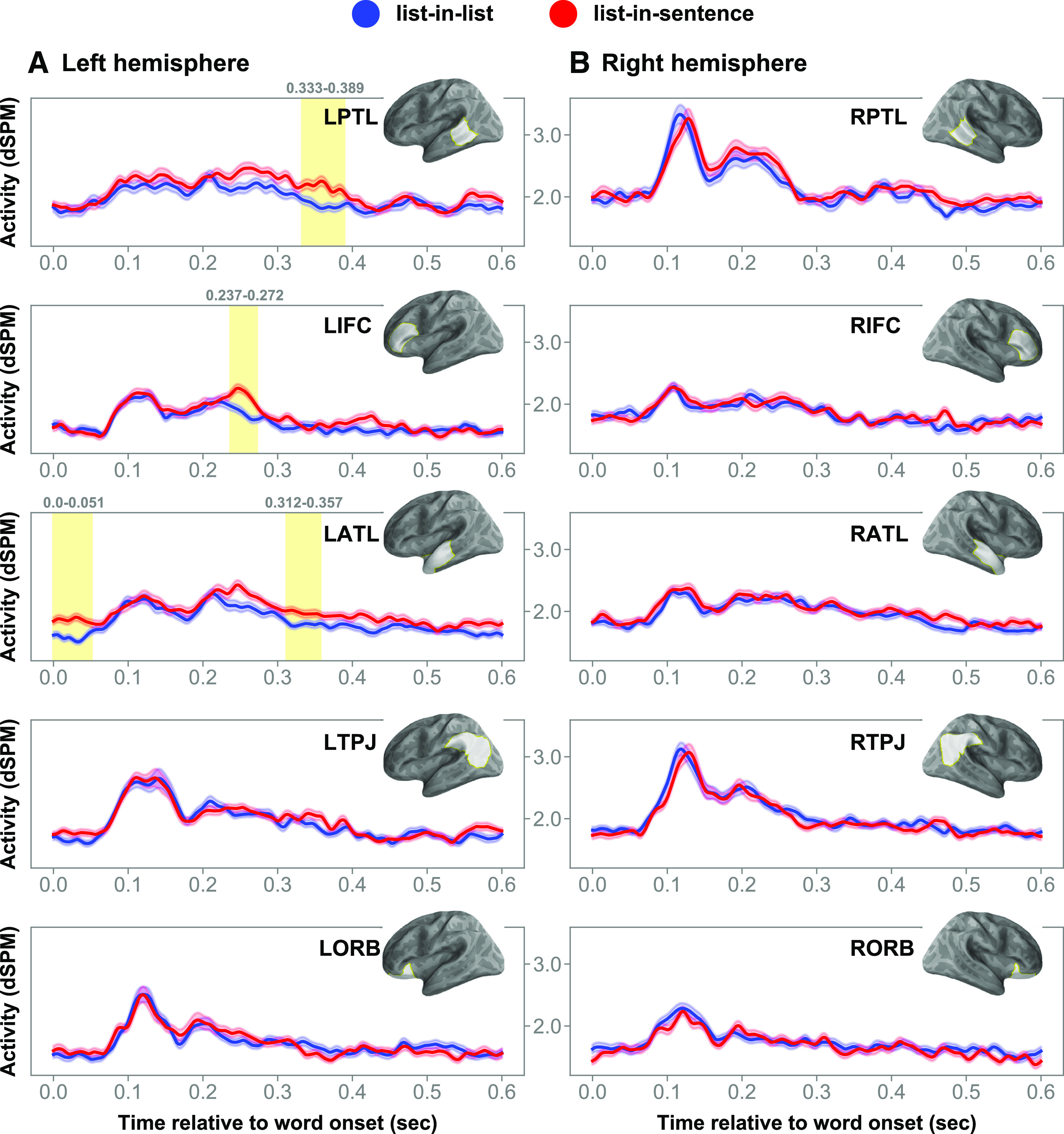
Main effects of structure in the left frontotemporal system. ***A***, ***B***, Charts of the time courses in the left (***A***) and right (***B***) hemispheres. The brain model indicates the ROI analyzed. Shaded regions in time series indicate cluster extent corresponding to FDR-corrected significant effects at *p* values <0.05. Error bars represent 1 within-subjects SEM (Loftus and Masson, 1994).

**Figure 5.**
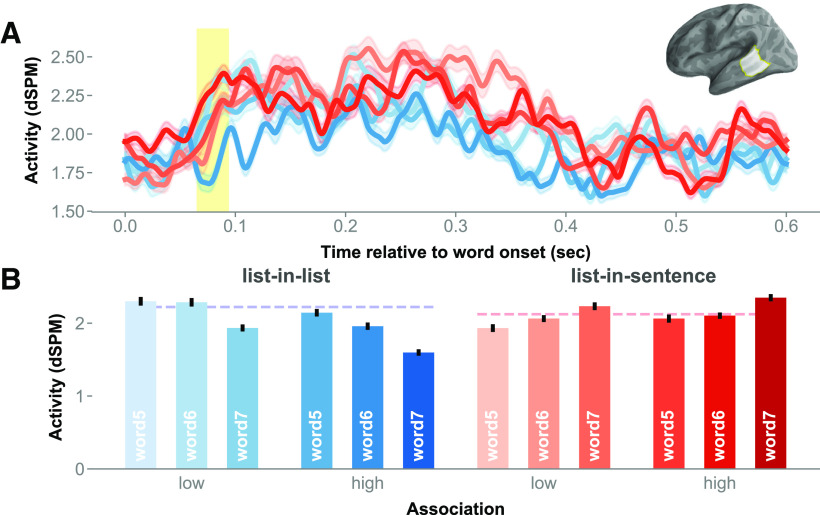
Structure-by-position interaction in the left PTL. ***A***, Chart of the activity time course in the left PTL, with cluster extent shaded in yellow. ***B***, Red-blue bar graphs, breaking down the interaction effect by the full design. Error bars represent 1 within-subjects SEM (Loftus and Masson, 1994).

In the left PTL, the cluster-based permutation test indicated that there are two effects, as follows: a main effect of structure (cluster mean *F* = 14.26, Cohen's *d* = 0.24, *p* = 0.004) and an interaction between structure and position (mean *F* = 7.44, Cohen's *d* = 0.21, *p =* 0.037). First, the main effect of structure corresponded to a cluster at ∼320–400 ms. Inspecting activity waveforms, lists in a structured expression increased PTL activity relative to lists in a longer list (Fig. 4). Second, the structure-by-position interaction corresponded to a cluster at ∼50–100 ms. In this cluster, word 5 from lists-in-lists and word 7 from lists-in-sentences elicited significantly stronger activity than word 7 in lists-in-lists (Fig. 5). In general, the activity level reduced as lists-in-lists progressed, while the opposite appears true for lists-in-sentences.

Turning to the left IFC, the structure effect was driven by a sharp increase in cortical signals elicited by structured lists relative to unstructured ones, as revealed by the permutation test in this region (mean *F* = 12.93, Cohen's *d* = 0.27, *p* = 0.028). This effect corresponded to a cluster spanning ∼230–280 ms post-word onset.

**Figure 6.**
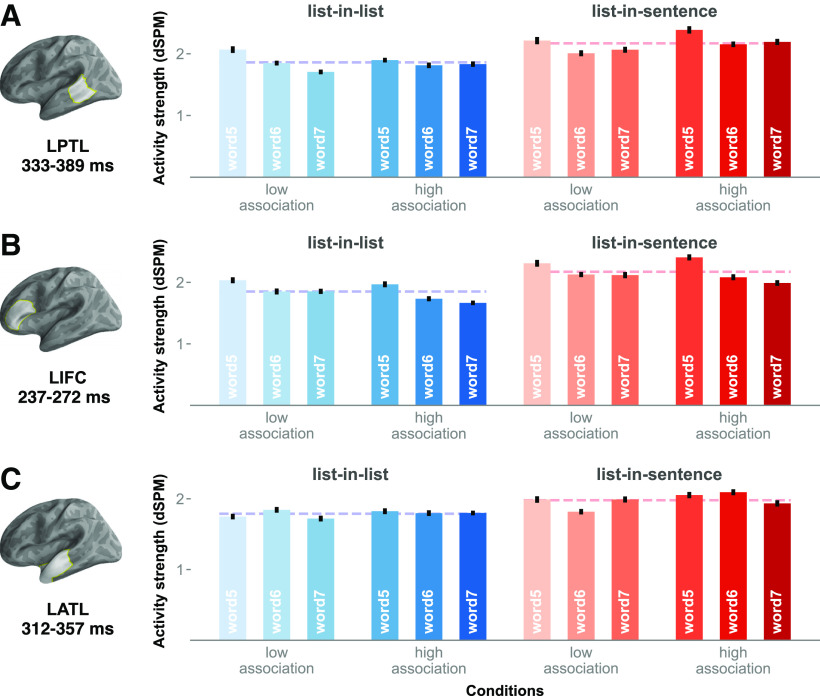
ROI activity associated with structure effects. ***A***–***C***, Averaged cluster activity by the full design in the left PTL (***A***), left IFC (***B***), and left ATL (***C***). Activity elicited by lists-in-lists is in blue, while that elicited by lists-in-sentences is in red. Dashed lines represent averaged cluster activity elicited by lists-in-lists and lists-in-sentences. Error bars represent 1 within-subjects SEM (Loftus and Masson, 1994).

Within the left ATL ROI, the permutation test revealed significant structure effects (mean *F* = 21.21, Cohen's *d* = 0.31, *p* = 0.003; mean *F* = 11.67, Cohen's *d* = 0.21, *p* = 0.024), which corresponded to two clusters. A cluster emerged very early at ∼0–50 ms post-word onset, while a second cluster emerged at 300–370 ms. In both of these clusters, lists-in-sentences elicited greater activity than lists-in-lists. Plotting left ATL activity time locked to each noun onset separately, the zero-onset cluster was driven by a waveform separation surrounding the onset of word 7; such a separation in activity waveforms was not observed surrounding the onsets of words 5 and 6. Thus, the early cluster was a result of averaging activity across the lists and did not occur consistently across the three positions. The main effects of structure were broken down by the full design in Figure 6.

In the right ORB, although there was no main effect of structure, structure and association did interact (mean *F* = 16.10, Cohen's *d* = 0.25, *p* = 0.027). This interaction was captured in a cluster at 170–210 ms post-word onset. ROI activity was higher for lists-in-lists with high association than with low association; this contrast in activity was not significant within lists-in-sentences.

**Figure 7.**
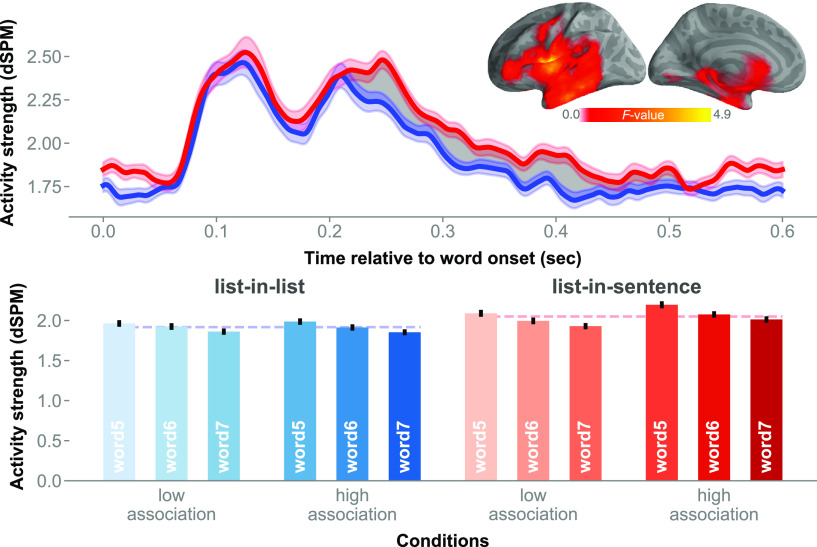
Left precentral and frontal opercular activity captured in whole-brain analysis. The shaded area in the time series marks the temporal extent of the cluster, while the brain model shows the spatial extent. The red-blue bar graphs break down the main structure effect by the full design.

In addition, we performed a complementary cluster-based spatiotemporal analysis across the whole brain. The analysis indicated a significant effect of structure (mean *F* = 1.40, Cohen's *d* = 0.16, *p* = 0.002): increased activity for structured lists relative to unstructured controls. A cluster extended temporally from ∼180 to 520 ms post-noun onset (Fig. 7). Spatially, the cluster extent was left lateralized, covering the left frontotemporal regions, portions of the left frontal operculum, the underlying left insula, as well as portions of the left precentral gyrus. This effect was not found in the right hemisphere.

### Neural effects of association

**Figure 8.**
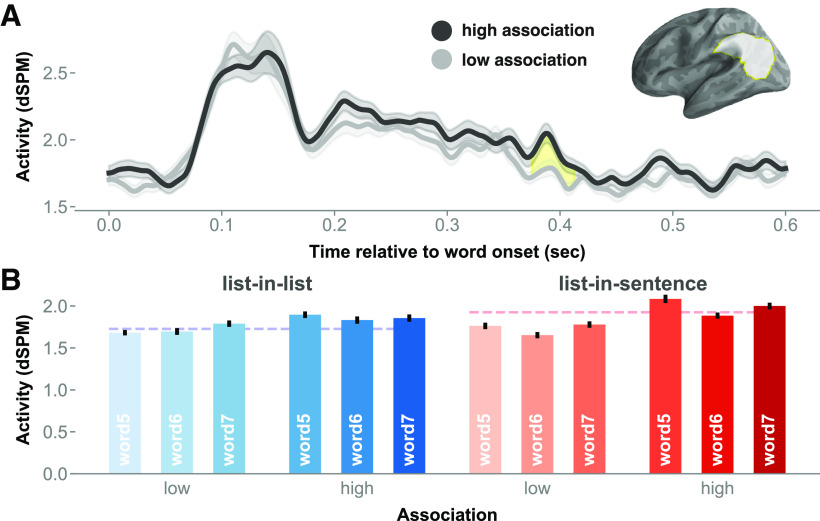
Main effect of association in the left PTL. ***A***, Chart of the activity time course in the left TPJ, with cluster extent shaded in yellow. ***B***, The red-blue bar graphs, breaking down the effect of association by the full design. Error bars represent 1 within-subjects SEM (Loftus and Masson, 1994).

A cluster extended from ∼370 to 420 ms after stimulus onset in the left TPJ. The permutation test in this region indicated a main effect of association (mean *F* = 9.45, Cohen's *d* = 0.25, *p* = 0.031; Fig. 8). Lists that were more strongly associated in terms of co-occurrence elicited stronger signals than lists in contexts that are relatively less associative. Examining the activity waveform, the cluster extent encompassed a peak in amplitude at ∼400 ms post-word onset, a timing that bore a semblance to that of the N400 event-related potential component. Averaged cluster activity time locked to each list item was plotted in bars in Figure 8*B*. There was no significant association-by-position interaction.

### Neural effects of position

**Figure 9.**
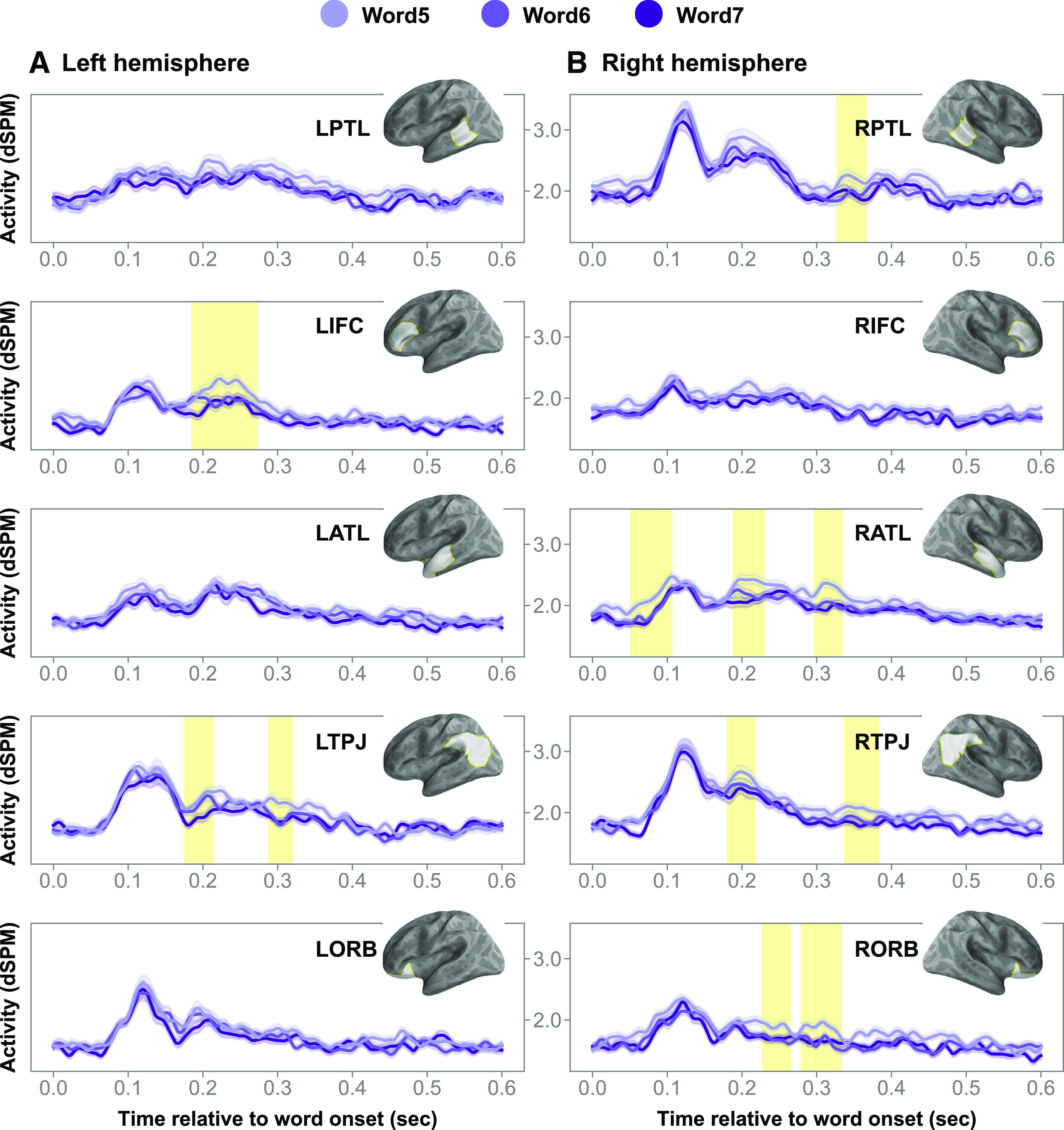
Main effects of position. ***A***, ***B***, Charts of the time courses in the left (***A***) and right (***B***) hemispheres. The brain model indicates the ROI analyzed. Shaded regions in time series indicate cluster extent corresponding to FDR-corrected significant position effects.

Across the ROI and spatiotemporal analyses, we also observed position effects (Fig. 9; mean *F* values = 5.07–8.13, Cohen's *d* values = 0.10–0.32, *p* values < 0.05). While this is not the main effect we sought to interpret, it is interesting to note that activity was greater for word 5 than words 6 and 7 across lists with and without structure. This pattern of activity was observed across a broad set of regions in both hemispheres.

Indeed, within a list embedded in a structured expression, word 5 (i.e., the first member of the list) is the first noun that the verb takes as a direct object (e.g., “The eccentric man hoarded lamps…”). This invites the question of whether the increase in activity we observed for lists-in-sentences were because of processes associated with the integration of the direct object with the verb (although obviously within our stimuli, the argument slot is not fully saturated until the rest of the conjunction is processed). Therefore, a possibility remains that the structure effects we observed above were largely driven by argument structure-related processes associated predominantly with word 5.

To investigate this possibility, we excluded word 5 and performed cluster-based permutation tests across all ROIs (and corrected for multiple comparisons accordingly) with the same test criteria outlined above. In other words, we performed a 2 × 2 × 2 ANOVA at each time sample in each ROI. We found that the structure effects held in the left PTL (mean *F* = 13.52, *p* = 0.01) and left ATL (mean *F* values > 9.93, *p* values < 0.05). The IFC effect on structure now only approached significance (mean *F* = 8.63, *p* = 0.08, uncorrected). The extents of corresponding clusters resemble their three-word counterparts in size and timing to a large extent. Thus, the reduction in IFC structure effect is likely a result of a reduction in statistical power from removing word 5.

## Discussion

The current MEG study used a novel approach—comparing lists with and without syntax—to identify candidate neural correlates of structure while controlling many related, hard-to-control stimulus properties. Within the combinatory network, the PTL, IFC, and ATL demonstrated sensitivity to the presence of structure. Our design allowed us to minimize contributions from the following sources: (1) word-level semantics (the same words occurred at the same time points within our structure contrast); and (2) local phrase combinatorics between critical list items (they were all plural, blocking noun–noun compounding). Further, our findings cannot be explained by general task demand differences (at least under the usual assumption that more effort leads to higher neural activity—here, activity was higher in cases where recall was facilitated). The main effects of structure corresponded to clusters at different time points, providing the temporal resolution lacking in previous hemodynamic work.

### Left posterior temporal lobe

We consider here several hypotheses about the role of the left PTL during combinatory processing. First, the PTL is thought to engage in lexical storage and retrieval (for review, see Lau et al., 2008). Word semantics are argued to be stored together with their associated syntactic information (Snijders et al., 2009; Rodd et al., 2010; Tyler et al., 2013; Matchin et al., 2017, 2019a,b). One of our critical findings is that we observed structure effects even when lexical items were identical: PTL activity was higher for lists-in-sentences compared with lists-in-lists. While the PTL contribution in lexico-syntactic access is evident from the literature, partaking in a syntactic tree appears to drive the left PTL above and beyond lexico-syntactic access.

Second, a recent MEG study demonstrated the early contribution of the PTL in syntactic composition, as evidenced by contrasting two cases in which semantic composition took place in both cases but syntactic composition only in one (Flick and Pylkkänen, 2020). =Our PTL finding is compatible with this account. Our study contrasts with that by Flick and Pylkkänen (2020) in that our critical list items did not compose with one another in either case, minimizing activity associated with local compositional semantics within our critical regions. The effect in the current study is a bit later, at ∼330–390 ms, while that in the study by Flick and Pylkkänen (2020) was at ∼200–230 ms. This timing difference possibly reflects distinct computations/processing stages, raising a question for future work on the function of the PTL.

The PTL was also recently proposed to engage in prediction about likely upcoming hierarchical syntactic structures (Matchin et al., 2017, 2019a). However, a possibility remains that semantic factors likely impact predictions online (Kuperberg and Jaeger, 2016). Moreover, studies reporting structural predictive effects (Matchin et al., 2019a) adopted a blocked design, which might encourage prediction. Cognizant of our fully randomized stimulus presentation, our results are compatible with this proposal.

Finally, the PTL is thought to engage in constituent structure building together with the IFC (Pallier et al., 2011) and/or sentence-meaning buildup together with other frontotemporal areas (Fedorenko et al., 2016). In our results, we observed a structure-by-position interaction only in this region (Fig. 5): words 5 from lists-in-lists and words 7 from lists-in-sentences elicited greater activity than words 7 in lists-in-lists. This finding is consistent with both proposals. However, it is worth pointing out that (1) we did not see (statistically significant) monotonic increases and, in fact, (2) activity decreased as lists-in-lists progressed. As to why this interaction preceded the main effect of structure, one could speculate that a time-resolved technique like MEG allowed us to detect PTL activity reflecting different stages/computations of syntactic processing. Thus, this raises new questions about the function of the left PTL across time.

### Left inferior frontal cortex

Activity increase associated with the presence of structure was observed in the IFC at ∼240–270 ms post-noun onset, an effect that saw a transient peak at ∼250 ms. This finding contrasts with the PTL finding, wherein increased activity had a more sustained quality starting at ∼220–390 ms and was only statistically significant at ∼330–390 ms. With caution, our interpretation of this activity pattern is that the left PTL and IFC likely carry out distinct functions with regard to syntactic processing, with both regions working in tandem during combinatory language comprehension (Tyler and Marslen-Wilson, 2008; Pallier et al., 2011; Griffiths et al., 2013).

### Left anterior temporal lobe

Meaning composition amounts to a cascade of processes at multiple levels of representation (e.g., syntactic, semantic, pragmatic). Thus, a body of work has sought to unpack the constituent processes and investigate the neural reflexes of meaning composition in two-word phrases (for review, see Pylkkänen, 2019). Two-word phrasal composition correlates with increased ATL activity ∼200 ms post-noun onset. Although originally compatible with a syntactic structure-building hypothesis (Bemis and Pylkkänen, 2011), subsequent studies suggest that this activity appears to be driven by conceptual combination (for review, see Pylkkänen, 2019). When comparing two expressions that have relatively parallel conceptual content but divergent syntactic combination, ATL remains insensitive (Flick and Pylkkänen, 2020). Preliminary results also show that the combinatorial steps underlying meaning composition in adjective–noun pairs are largely insensitive to syntactic structure (Parrish and Pylkkänen, 2019; Kim and Pylkkänen, 2020).

Given our design, wherein local phrasal composition within the critical lists was blocked, one would predict the absence of a combinatory left ATL effect. Instead, our left ATL results showed a main effect of structure. This finding shows the left ATL in a new light, opening up a new research question about the function of this region. To the extent that combinatory syntax and semantics can be dissociated, a pertinent question, then, is whether the ATL activity observed in our study was reflective of structure or sentence-level semantic interpretation. In sum, the presence of structure effect and the absence of an association effect within the left ATL rule out explanations in terms of bottom-up lexical access and local semantic composition.

### Left temporoparietal junction: associative semantics

We found the following main effect of association in the left TPJ: stronger association among list items and contexts elicited stronger activity. Mindful of the sensitivity of the N400 to association, the directionality of our TPJ effect—peaking ∼400 ms post-stimulus onset—was ultimately opposite to what would be expected for the N400. When words are primed with associated words, N400 amplitude is expected to lower (i.e., more negative; Rhodes and Donaldson, 2008; Ortu et al., 2013; for review, see Kutas and Federmeier, 2011). By contrast, we saw increased source amplitude for words in more associative contexts. This suggests that our TPJ finding is not just a source-localized N400 effect.

Importantly, left TPJ activity increased as a function of semantic association strength regardless of structure. A tentative hypothesis might be that, as levels of association strength increased, the brain might attempt to “make sense” out of the plural nouns. This computation might take the form of (a combination of) semantic composition (Mollica et al., 2020) and chunking (Christiansen and Chater, 2016). For example, the highly associative list “pianos, violins, guitars” might result in a grouping of a “musical instrument” set compared with a less associative list “lamps, doll, guitars.” Note, though, that although a chunking procedure would reduce working memory load, our finding is not consistent with studies showing TPJ activation for memory-intensive dependencies (Novais-Santos et al., 2007; Meyer et al., 2012).

In all, the left TPJ is the only region that showed sensitivity to our association contrast and, critically, not to the structure contrast. This suggests a more conceptual–semantic function as opposed to a syntactic one for the TPJ, which is consistent with previous proposals (Pallier et al., 2011; Matchin et al., 2019a,b).

### Limitations and future directions

Throughout the article, we have alluded to the fact that a confound exists in our design: lists-in-sentences introduce a global semantic context that describes a scenario, while lists-in-lists do not. A corollary is that perhaps just comparing identical words within a structured–unstructured pair might not be a sufficient control of word semantics, since the interpretation of a word is context dependent. While our design does not control global semantics, our design succeeds in minimizing bottom-up contributions from individual word meanings and phrasal composition.

Therefore, the current paradigm carves out the hypothesis space for future work to distinguish effects of sentence-level message from those of syntactic structure. This paradigm can also be extended to other syntactic categories such as adjectives and verbs. It is possible that our set of results does not generalize beyond noun lists. Thus, a straightforward follow-up would be to extend this paradigm to such other syntactic categories and draw up lists accordingly (e.g., “the big blue wooden ball”).

### Conclusion

The present study leveraged word lists, which are typically used as unstructured controls, to deconfound syntax from semantics. We found that the left PTL, left IFC, and left ATL showed sensitivity to structure independent of lexico-conceptual semantics and local combinatorics. A structure-by-region interaction indicated that the observed difference between lists-in-lists and lists-in-sentences was largest in the left PTL. While explanations in terms of the global semantics of the sentences cannot yet be ruled out, this pattern of results allowed us to rule out explanations in terms of lexical semantics and local semantic composition. Our left TPJ finding supports a conceptual-semantic role for the region. These findings contribute a piece to our relatively coarse understanding of the extent to which syntactic and semantic processes could be teased apart at the level of brain regions, as well as providing the temporal resolution that hemodynamic work lacks.
